# TIMP-1 enhancer sequence – real or bacterial?

**DOI:** 10.1038/sj.bjc.6601353

**Published:** 2003-10-28

**Authors:** I M Clark, D A Young, D R Edwards

**Affiliations:** 1 School of Biological Sciences, University of East Anglia, Norwich NR4 7TJ, UK

**Sir**,

We have read with interest the recent paper in the *British Journal of Cancer* (Wang *et al*, 2003). Our groups have worked on TIMP-1 gene regulation for a number of years, including a dissection of the gene promoter in both human and mouse.

The 18 bp HTE-1 enhancer element used in this paper (and others referenced therein) is described to be in intron 1 of the human TIMP-1 gene. However, a search of sequences from our own human and mouse TIMP-1 genomic clones, or indeed a BLAST into the human genomic sequence databases, including the X-chromosome sequences containing the TIMP-1 gene, do not reveal a perfect match for HTE-1. The best match in the human genome, for the first 16 base pairs of HTE-1, is in chromosome 17 (though outside of the TIMP-2 gene which lies on this chromosome). It is with some concern that we note that the HTE-1 sequence is matched in its entirety within the *E. coli* genome.

The authors have cloned an IL-10-responsive DNA-binding protein on the basis of its binding to the HTE-1 sequence, and explored its function. However, the interpretation of the data demonstrating that overexpression of the HTE-1-binding protein induces TIMP-1 expression (Figure 11) is made more difficult if the HTE-1 sequence is not within the TIMP-1 gene.

We would very much welcome the authors' input on these matters.
